# The Impact of Tobacco Use on COVID-19 Outcomes: A Systematic Review

**DOI:** 10.1155/2022/5474397

**Published:** 2022-01-20

**Authors:** Jessica Baker, Nandita Krishnan, Lorien C. Abroms, Carla J. Berg

**Affiliations:** ^1^Department of Global Health, Milken Institute School of Public Health, The George Washington University, Washington, DC, USA; ^2^Department of Prevention and Community Health, Milken Institute School of Public Health, The George Washington University, Washington, DC, USA; ^3^George Washington University Cancer Center, George Washington University, Washington, DC, USA

## Abstract

**Introduction:**

Tobacco use increases risks for numerous diseases, including respiratory illnesses. We examined the literature to determine whether a history of tobacco use increases risks for adverse outcomes among COVID-19 patients.

**Methods:**

We conducted a systematic search of PubMed, LitCovid, Scopus, and Europe PMC (for preprints) using COVID-19 and tobacco-related terms. We included studies of human subjects with lab-confirmed COVID-19 infections that examined tobacco use history as an exposure and used multivariable analyses. The data was collected between March 31^st^, 2020, and February 20^th^, 2021. Outcomes included mortality, hospitalization, ICU admission, mechanical ventilation, and illness severity.

**Results:**

Among the 39 studies (33 peer-reviewed, 6 preprints) included, the most common outcome assessed was mortality (*n* = 32). The majority of these studies (17/32) found that tobacco use increased risk, one found decreased risk, and 14 found no association. Tobacco use was associated with increased risk of hospitalization in 7 of 10 studies, ICU admission in 6 of 9 studies, mechanical ventilation in 2 of 6 studies, and illness severity in 3 of 9 studies. One study found that tobacco use history increased risk of pulmonary embolism in COVID-19 patients. Tobacco use was found to compound risks associated with diabetes (*n* = 1), cancer (*n* = 2), and chronic liver disease (*n* = 1).

**Conclusion:**

There is strong evidence that tobacco use increases risks of mortality and disease severity/progression among COVID-19 patients. Public health efforts during the pandemic should encourage tobacco users to quit use and seek care early and promote vaccination and other preventive behaviors among those with a history of tobacco use.

## 1. Introduction

The emergence of COVID-19 in the last two years represents a grave threat to global health. Researchers have worked rapidly to better understand the risk factors associated with COVID-19, in order to prevent infections and reduce the severity of illness in those infected. Initially considered a respiratory infection, COVID-19 is now understood to be a systemic infection, which can cause a wide variety of complications throughout the body. SARS-CoV-2 commonly targets Angiotensin-Converting Enzyme 2 (ACE2) receptors in a host's lungs and downregulates ACE2 expression [1]. ACE2 receptors are part of the Renin-Angiotensin System (RAS), which plays a complex role in regulating cardiovascular, renal, and metabolic functions. Thus, individuals living with comorbidities associated with RAS dysregulation (e.g., kidney disease, diabetes mellitus, and cardiovascular and pulmonary diseases) are at increased risk of severe COVID-19 [2, 3].

Tobacco use is a well-documented risk factor for hypertension, lung disease, cardiovascular disease, cancer, and diabetes and is a leading cause of chronic disease and death worldwide [4]. Additionally, cigarette smoking is a known risk factor for contracting infectious respiratory diseases such as influenza, tuberculosis, and MERS, as well as exacerbating the outcomes of those infections [5, 6]. Research has found that smoking increases ACE2 receptors in the lungs, indicating that smokers may be more vulnerable to infection [7, 8]. While the exact mechanisms of the interaction between tobacco and the RAS are complex, evidence shows that nicotine itself dysregulates the RAS [2]. Since preexisting dysregulation of the RAS and its associated comorbidities worsen the severity and outcomes of COVID-19, understanding the impact of tobacco use on COVID-19 outcomes could significantly impact both prevention and treatment of COVID-19.

Given the explosion of research on SARS-CoV-2, systematic reviews of the literature are needed to inform evidence-based policies and practices to reduce the population impact of COVID-19. Since tobacco is a known risk factor for other infectious diseases, several reviews have focused on its impact on COVID-19 (see Table 1). The earliest review of COVID-19 and smoking that the authors are aware of was completed in March 2020 and provided initial findings from January to February 2020 [9]. Reviewing five relatively small studies, all from China, the study concluded that tobacco smoking likely had a negative impact on COVID-19 disease progression and outcomes. Several reviews followed in early 2020, all of which relied heavily on relatively small studies from China [10–12]. In June 2020, Gulsen et al. conducted a systematic review and meta-analysis which included 16 studies, 14 of which were from China and 2 of which were from the United States. This study more firmly concluded that a history of smoking is associated with severe COVID-19 [13].

As more research was published in the summer and fall of 2020, reviews were able to include studies from a wider range of countries. One meta-analysis of 10 studies published in October 2020 included studies from Italy, Thailand, and the United Kingdom and specifically analyzed the association between smoking and mortality in COVID-19 patients [14]. However, this study drew only from one database (PubMed) and did not examine outcomes beyond mortality. Another review and meta-analysis included 47 peer-reviewed articles published between January and late May 2020 and analyzed the impact of smoking behavior on COVID-19 progression and mortality [15]. This may be the largest review to date specifically focused on tobacco use and COVID-19 outcomes, and it concluded that smoking is an independent risk factor for COVID-19 severity and death. However, some of the included studies had very small numbers of smokers (studies were excluded when the number of smokers was “zero or omitted”) and the study did not have a requirement regarding how COVID-19 was diagnosed. A literature review exploring the relationship between tobacco-related comorbidities and COVID-19 outcomes was published in November 2020 [16]. This review included 23 studies published between January and September 2020. Although this was not a systematic review, it did find an association between tobacco use, cardiopulmonary diseases, and negative COVID-19 outcomes.

Finally, the most recent review at the time of this writing included 40 studies published up to December 12, 2020 [17]. This systematic review and meta-analysis found that current and former smoking increased the risk of COVID-19 severity and death. Of the studies included, 19 were from in China, 12 were from the US, and the rest were from other countries. Although this review assessed the quality of included studies, it did not have requirements regarding how COVID-19 was diagnosed or the number of smokers a study had to include in its sample.

The current study was aimed at identifying the impact of tobacco use on COVID-19 outcomes. Specifically, this review was aimed at adding to the existing literature in three main ways: (1) studies included had a sample of 30 or more current and former smokers, (2) only laboratory-confirmed cases of COVID-19 were included, and (3) to the extent possible, this review explored specific outcomes beyond general disease severity and mortality. Additionally, since early studies have already been heavily reviewed, we only included studies that completed data collection after March 31, 2020. Findings from this study can improve our understanding of the role of tobacco use as a risk factor for SARS-CoV-2 and facilitate our ability to draw actionable conclusions based on the available data.

## 2. Methods and Materials

This systematic literature review identified articles through keyword searches using the following databases: PubMed, Scopus, LitCovid, and Europe PMC (for preprints). The full list of search terms can be found in the Appendix and includes three themes: (i) terms related to tobacco such as “tobacco,” “smoking,” and “vaping”; (ii) terms related to COVID-19, including “SARS-CoV-2” and “novel coronavirus”; and (iii) terms related to outcomes, such as “mortality” and “hospitalization.”

Inclusion criteria for studies were as follows: (i) written in English; (ii) collected data between March 31, 2020, and February 20, 2021; (iii) sample size of 30 or more current or former tobacco users; (iv) lab-confirmed COVID-19 diagnosis; (v) assessed history of tobacco use as an exposure; and (vi) used multivariable analysis. Studies that were published in other languages; completed data collection before March 31, 2020; focused on animal subjects; relied on self-reported or symptom-diagnosed COVID-19; had fewer than 30 current or former tobacco users; conducted only univariate or descriptive analyses; or were unrelated to tobacco use were excluded. Case studies, ecological studies, in vitro and in silico studies, and Mendelian randomization studies were also excluded. Other reviews were included in the first stage of the search to screen for references and were then excluded.

One researcher (JB) conducted an initial screening of titles and abstracts to eliminate articles that did not meet the inclusion criteria. Two researchers (JB and NK) reviewed the remaining titles and abstracts more closely to further eliminate articles that did not meet inclusion criteria. JB and NK then conducted a full-text review of included articles. One researcher (JB) conducted the data extraction for final analysis.

Researchers used a wide variety of metrics. Lab-confirmed COVID-19 was primarily based on a positive result on an RT-PCR test. However, a few studies confirmed COVID-19 using other lab tests such as antibody or serum tests or CT radiology scans. Diagnoses based on symptoms were excluded. The type of tobacco use discussed in almost all the studies (*n* = 38) was combustible smoking. One study focused on smokeless tobacco. Tobacco use was most commonly reported as ever vs. never use (*n* = 15) or never/former/current use (*n* = 13). Two studies analyzed differences in pack-years smoked, where pack-years referred to the number of packs smoked per day times the number of years smoked.

Finally, “outcome” was defined as what happened after a person was infected with COVID-19. Outcomes for this analysis included the following: mortality (*n* = 32), hospitalization (*n* = 10), ICU admission (*n* = 9), mechanical ventilation (*n* = 6), and severity of illness (*n* = 9). Studies defined “severity” in different ways. For example, Adrish et al. [18] defined severity on the basis of pneumonia and hypoxia status, while Mendy et al. [19] defined severity as admission to ICU or death during hospitalization. Studies focused on the incidence or transmission of COVID-19 were excluded.

## 3. Results

**Figure 1:**
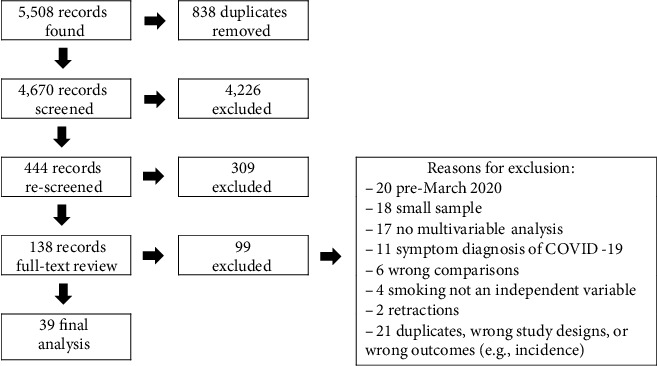
PRISMA chart.

The initial search, done on February 20, 2021, identified 5,980 articles. Of these articles, 472 were published before December 2019 and removed from the Scopus search, leaving 5,508 citations. The searches were uploaded to Covidence for screening. Covidence identified 838 duplicates, which were reviewed for confirmation and then removed, leaving 4,670 articles to screen. The breakdown of the results can be seen in the PRISMA chart in Figure 1.

In the first round of title and abstract screening, 4,223 articles were excluded. Many of these (*n* = 1,743) were thematically irrelevant. For example, they discussed adapting medical procedures in the context of COVID-19 or were otherwise unrelated to tobacco use (*n* = 1,056), focused on behavior or socioeconomic impacts (*n* = 694), focused on diseases other than COVID-19 (*n* = 420), or focused on incidence risk (*n* = 219) or were in silico, in vitro, or animal studies (*n* = 148). Two of the authors (JB and NK) reviewed the titles and abstracts of the remaining 447 articles. The second round of review eliminated another 309 articles, including ecological studies, case studies, reviews, and commentaries. The remaining 138 articles were included in the full-text review. Of these, a further 99 were excluded for the following reasons: only included data collected before March 31, 2020 (*n* = 20), sample sizes included fewer than 30 current or former tobacco users (*n* = 18), did not include smoking in a multivariable analysis (*n* = 17), included cases of COVID-19 not confirmed by lab results (*n* = 11), did not analyze the relationship between tobacco use and COVID-19 outcomes (*n* = 6), and did not include smoking as an independent variable in the analysis (*n* = 4). Additionally, 21 studies were excluded as they were duplicates, used the wrong study designs, or contained the wrong outcomes (e.g., incidence of COVID-19 rather than severity), and a further 2 studies were excluded because the articles were retracted.

In total, 39 studies were included in this review. At the time of the initial review, 29 of these studies were peer-reviewed manuscripts and 10 were preprints. Four preprints were subsequently published and are included in the peer-reviewed count. The characteristics of the studies can be seen in Table 2, with peer-reviewed and preprint studies listed separately. Studies had sample sizes that ranged from 101 to 406,793, with history of tobacco use sample sizes ranging from 35 to 24,484. Fourteen countries were represented by the studies, with 45% of studies done in the United States. Twenty-six studies used retrospective observational cohort designs, 8 used prospective/longitudinal study designs, 3 used case-control designs, and 2 used cross-sectional designs.

### 3.1. Mortality

Mortality was the most common outcome used across studies to assess the impact of tobacco use on COVID-19. Out of the 39 studies, 32 assessed the association between tobacco use and mortality. Of the 28 peer-reviewed articles that assessed mortality, 12 found no significant association, 15 found an increased risk of death, and one found a decreased risk of death. Of the 4 preprints that assessed mortality, 2 did not find a significant association and 2 found that tobacco use increased the risk of death. Seven articles did not report on mortality. The one article that found a significantly decreased risk of death came from Mexico, using Ministry of Health data [20]. However, in a univariate analysis, the authors found a significant risk in men but not in women, which they suggested could be due to a dose-response relationship, as men are much heavier smokers than women.

A few studies focused on specific populations. Two studies among cancer patients with COVID-19 both found that tobacco use significantly increased the risk of mortality, and this was most common for patients with respiratory tract cancers [21, 22]. A study on chronic liver disease patients with COVID-19 also found a significantly increased risk in this population [23].

Regarding mortality data, not all studies divided their data in the same way. Some studies analyzed former and current tobacco users separately. There was a trend in studies finding more significant risk in former smokers compared to current smokers. One factor that could explain this trend is that the pool of former smokers tended to be much larger than current smokers—often 3-5 times larger. Likewise, in some studies, the number of patients who died was very small. Even with an initial pool of 100 smokers, some studies only observed 2 or 3 deaths within that group, making statistical analysis difficult. Some of the studies that did not find a significant association in multivariable analysis found increased mortality risk in univariate analysis [18, 24].

One study identified a dose-response effect based on pack-years, with those with a history of 30 pack-years or more having a significantly higher risk of death than those with a lower pack-year history [25]). As suggested by the Parra-Bracamonte et al. study, there might be a dose-response relationship between tobacco use history and COVID-19 mortality risk.

### 3.2. Disease Severity and Progression

In addition to mortality, 23 studies (19 peer-reviewed and 4 preprints) assessed a variety of other indicators for disease severity and progression (see Table 2). Eight (7 peer-reviewed and 1 preprint) studies found no significant association between tobacco use and the COVID-19 outcomes they measured. However, 15 (12 peer-reviewed and 3 preprints) studies found a significantly higher risk of disease severity or progression for people with a history of tobacco use. None of these 23 studies reported a significantly decreased risk.

Studies defined severity and progression in a wide variety of ways. The most common measures were hospitalization (*n* = 10), ICU admission (*n* = 9), the need for mechanical ventilation (*n* = 6), and severity (*n* = 9). Across the studies, tobacco smokers were found to be at significantly higher risk of hospitalization (*n* = 7) and ICU admission (*n* = 6) than nonsmokers. Two studies found smokers at increased risk of mechanical ventilation, and three found smokers to be at increased risk of severe or critical illness.

A few other outcomes were also assessed. One study found that smokers were more likely to have chest X-ray abnormalities 12 weeks after hospitalization, which correlated with longer hospital stays and longer recovery times [26]. Another study also indicated that smokers may be at risk of longer hospital stays or longer disease duration, but results were not conclusive [27]. One study found that smokers were at higher risk of pulmonary embolism, an outcome that is associated with higher mortality [28]. And finally, one study found that smokers were at increased risk of losing their sense of smell [29].

Multiple studies noted the high correlation between a history of smoking and increased comorbidities, as well as the association between multiple comorbidities and increased risk of negative COVID-19 outcomes [25, 30–34]. One study that compared smokers, diabetics, and diabetics who smoke found significantly worse outcomes, including increased risk of death, for the diabetics who smoke compared to the other groups [27]. This finding highlights the crucial interaction between noncommunicable and infectious diseases.

## 4. Discussion

The explosion of research on COVID-19 may best be seen in NIH's LitCovid database, which now has over 124,000 articles on the subject and sees over 2,000 new articles each week. This unparalleled rate of research offers a wealth of information, but much of the information is incomplete as we continue to learn about COVID-19 and its impacts. This review sought to organize and update existing knowledge and add to what has been done using more rigorous inclusion criteria.

The findings of this review indicate that smokers, including those who have quit, are at greater risk of developing severe COVID-19 illness compared with never smokers. As tobacco use and COVID-19 both dysregulate the RAS, ever-smokers who contracted COVID-19 were more likely to experience hospitalization, ICU admission, and death. Furthermore, the interaction of COVID-19 and smoking history was found to significantly increase the risk posed to individuals living with chronic diseases such as chronic liver disease, diabetes, and cancer [21, 23, 27], a finding that has not been documented in prior reviews.

In conducting this review, a number of future research directions were identified. First, several other outcomes were seen in studies that did not meet our inclusion criteria and thus were not included in this review. However, these outcomes, including stroke, blood clots, heart attacks, sepsis, mental illness, and neurological outcomes, deserve further study. Second, evidence suggests other specific patient populations with a history of smoking, such as people living with asthma and COPD, may also be at increased risk. Third, research on COVID-19 among younger populations and the impact of vaping on COVID-19 is still very limited. No studies on vaping met our inclusion criteria. Considering the growing evidence of long-lasting symptoms, neurological side effects, and other disabilities related to COVID-19, more information on how COVID-19 impacts young adults is urgently needed. Additionally, while a few articles found that noncombustible nicotine use such as hookah and vaping was correlated with increased incidence of COVID-19 [35, 36], little research has examined the association between these forms of tobacco use and COVID-19 outcomes. Finally, evidence suggests a dose-response relationship between tobacco use and health outcomes. Therefore, dosage should be considered more widely in analyses of the impact of tobacco use on COVID-19 outcomes.

### 4.1. Strengths and Limitations

This systematic review focused on the impact of tobacco use on COVID-19 outcomes, including hospitalization, ICU admission, mechanical ventilation, and mortality. It achieved this goal by using appropriate search terms to gather available research, applying a rigorous methodical approach, and consolidating the findings into useful results to inform public health policy. However, there are limitations. With over 2,000 articles on COVID-19 published each week, any review, including this one, is inherently behind the latest research. In an effort to mitigate this limitation, preprints were included in the analysis. However, preprints have their own limitations, as they are not yet peer-reviewed. Recognizing this limitation, we presented results of preprints separately in Table 2. While this review was able to include studies from a variety of countries, it was still limited to English-language articles, excluding work done in Chinese, Italian, Spanish, and other languages. Additionally, the wide range of measurements used across studies made comparison difficult. For example, while some studies separated current vs. former smokers, others combined all participants with a history of tobacco use into one pool. While this may solve the problem of small samples, it may also dilute the results. Perhaps most importantly, the majority of studies relied on medical records, which may be missing data or contain incomplete histories. Lowe et al. [25] point out that patients with complete records are more likely to be wealthy and regularly access healthcare, potentially leading to an underestimate of the impact of tobacco use on COVID-19 outcomes.

## 5. Conclusions

Tobacco use increases the risk of severe illness, hospitalization, and mortality due to COVID-19. Public health efforts to minimize these outcomes should include raising awareness of these risks, promoting the uptake of and increasing access to tobacco cessation, encouraging tobacco users to seek care early in their illness, and prioritizing vaccination for those with a history of tobacco use. Some states within the United States have prioritized tobacco users in vaccination efforts. Given the findings of this review, we recommend expanding this policy.

## Figures and Tables

**Table 1:** Early and later reviews on tobacco use and COVID-19.

Author	Date	Studies	Conclusions	Notable limitations
Vardavas et al.	March 2020	5 from China	Smoking likely had a negative impact on COVID-19 disease progression and outcomes	Very small sample, very early data
Berlin et al.	April 2020	6 from China	More evidence is needed if tobacco use may be a risk factor for both transmission and negative outcomes	Small sample, early data
Farsalinos et al.	May 2020	13 from China	Smokers are underrepresented among hospitalized patients; former smokers have higher odds of adverse outcomes than current smokers; nicotine may be protective	Relies on early data exclusively from China; unadjusted for confounding factors including sociodemographic factors
Gulsen et al.	June 2020	14 from China, 2 from Italy	History of smoking is associated with severe COVID-19	Relies heavily on early data from China; classifications of both smokers and COVID-19 patients varied
Patanavanich et al.	Sept 2020, preprint	47 from 16 countries	Smoking is an independent risk factor for COVID-19 severity and death	Does not include research published after May 25, 2020; includes studies with very small (>0) smoking populations; no requirement regarding COVID-19 diagnosis
Salah et al.	Oct 2020	10 from various countries (China, UK, Thailand)	Smoking doubles the risk of mortality in COVID-19 patients	Used only PubMed; included mortality as an outcome
Gupta et al.	Nov 2020	23 from various countries (China, US, Italy)	Tobacco use is associated with comorbidities which increase the likelihood of negative COVID-19 outcomes	Literature review; not systematic
Umnuaypornlert et al.	Feb 2021	40 from 8 countries	Tobacco use increases the risk of disease severity and death in COVID-19 patients	No requirement regarding COVID-19 as diagnosis; no minimum number of smokers to be included in studies

**Table 2:** Study characteristics and results.

Author	Month and year	Location	Study design	Participants	Smokers	Mortality	Hospitalization	ICU admission	Mechanical ventilation	Severity
Parra-Bracamonte, G.M. et al.	Dec 2020	Mexico	Retrospective cohort	331,298	24,484 (7.4%)	OR = 0.931, *p* = 0.0151	∗	∗	∗	∗
Ioannou, G.N. et al.	Sept 2020	USA	Longitudinal multicenter cohort	10,131	5,212 (51.4%)	NS	NS	∗	NS	∗
Ho, K.S. et al.	Dec 2020	USA	Retrospective multicenter cohort	9,991	2,212 (22.1%); 1,279 (26.1%) hospitalized	NS	NS	NS	NS	∗
Bello-Chavolla, O.Y. et al.	June 2020	Mexico	Retrospective cohort	20,804	1,815 (8.7%)	NS	OR = 1.2, *p* < 0.05	NS	∗	∗
Pérez-Sastré, M.A. et al.	July 2020	Mexico	Retrospective cohort	16,752	1,340 (8%)	NS	NS	Prev ratio = 0.90, *p* < 0.05	∗	∗
Lowe, K.E. et al.	Jan 2021	USA	Retrospective multicenter cohort	7,102	1,082 (15.2%)	aOR = 1.89, *p* < 0.05 (>30 pkyr), NS for less	aOR = 1.41 (10-30 pkyr) & 2.25 (+30 pkyr), *p* < 0.05	aOR = 1.55 (10-30) and 1.69 (+30), *p* < 0.05	∗	∗
Lohia, P. et al.	Feb 2021	USA	Retrospective multicenter cohort	1,871	704 (37.6%)	OR = 1.26, *p* = 0.02; aOR NS	∗	aOR = 1.25, *p* = 0.03	NS	∗
Hamer, M. et al.	July 2020	UK	Prospective cohort	387,109 (760 with COVID-19)	406 (53.4%) (37k/9.7% current smokers in whole cohort)	∗	aRR = 1.36, *p* < 0.05	∗	∗	∗
Vilches-Moraga, A. et al.	Dec 2020	UK & Italy	Multicenter observational cohort	831	361 (43.4%)	∗	∗	∗	∗	∗
Kim, D. et al.	Sept 2020	USA	Retrospective observational multicenter cohort	867	354 (40.7%)	HR = 2.99, *p* = 0.001 (current smokers), NS for former	∗	∗	∗	NS
Adrish, M. et al.	Oct 2020	USA	Retrospective cohort	1,173	336 (28.6%)	NS	∗	∗	47% smoke vs. 37% nonsmoke, *p* = 0.005	47% smoke vs. 37% nonsmoke, *p* = 0.003
Di Castelnuovo, A. et al.	Oct 2020	Italy	Retrospective observational multicenter cohort	3,894	319 (8.2%)	NS	∗	∗	∗	∗
Raines, A.M. et al.	Feb 2021	USA	Retrospective cohort	440	250 (56.8%)	aOR = 2.28, *p* < 0.01	∗	∗	∗	∗
Islam, M.Z. et al.	Oct 2020	Bangladesh	Retrospective single center cohort	1,016	185 (18.2%); 40 smokeless tobacco (3.9%)	OR = 3.516, p < 0.05, RR = 3.33; aOR for smokeless NS	∗	∗	∗	NS
Saurabh, S. et al.	Jan 2021	India	Prospective unmatched case-control study	911	80 (8.8%); 139 (15.3%) smokeless tobacco	NS	∗	∗	∗	NS
Soares, R.C.M. et al.	Sept 2020	Brazil	Retrospective multicenter cohort	10,713	209 (1.95%)	NS	OR = 2.91, *p* < 0.001	∗	∗	∗
Alharthy, A. et al.	Oct 2020	Saudi Arabia	Retrospective single center cohort	352	174 (49.4%)	OR = 3.0, *p* = 0.025	∗	∗	∗	∗
Abohamr, S.I. et al.	Nov 2020	Saudi Arabia	Retrospective case series	768	160 (20.8%)	OR = 7.018, *p* = 0.001	∗	OR = 2.991, *p* = 0.001	∗	∗
Garassino, M.C. et al.	July 2020	8 countries	Cross-sectional & longitudinal multicenter observational cohort	196	159 (81.1%)	OR = 3.18, *p* < 0.05	∗	∗	∗	∗
Abbas, H.M. et al.	Sept 2020	Iraq	Cross-sectional observational follow-up	284	141 (49.6%)	HR = 1.36, *p* = 0.014 (smoke only); HR = 1.66, *p* < 0.01 (smoke + diabetes)	∗	∗	∗	NS
Ullah, A.D. et al.	Oct 2020	UK	Retrospective single-center cohort	212	126 (59.4%)	NS	∗	∗	∗	∗
Ragab, E. et al.	Nov 2020	Egypt	Retrospective cohort	240	116 (48.3%)	∗	∗	∗	∗	OR = 3.31, *p* = 0.005
Chen, L. et al.	Aug 2020	China	Retrospective multicenter cohort	1,859	111 (6%)	HR = 1.84, *p* = 0.009	∗	∗	∗	∗
Killerby, M.E. et al.	June 2020	USA	Retrospective cohort	531	91 (17.1%)	∗	aOR = 2.3, *p* < 0.05	∗	∗	∗
Chetboun, M. et al.	Sept 2020	6 countries	Retrospective multicenter cohort	1,461	83 (6.5%) (data for 1,275)	NS	∗	∗	NS	∗
Chand, S. et al.	Oct 2020	USA	Retrospective case series	300	67 (22.3%)	RR = 1.35, *p* = 0.01	∗	∗	∗	∗
Palaiodimos, L. et al.	July 2020	USA	Retrospective cohort	200	65 (32.5%)	NS	∗	∗	∗	OR = 2.1, *p* = 0.022
Badr, O. et al.	Feb 2021	Saudi Arabia	Retrospective case-control	159	61 (38.4%)	NS	∗	∗	∗	∗
Sapienza, L,G. et al.	Jan 2021	USA	Retrospective cohort (2 sites)	154	56 (36.4%)	OR = 5.47, *p* = 0.008	∗	∗	∗	∗
Bellan, M. et al.	Nov 2020	Italy	Retrospective multicenter cohort	1,697 (407 assessed for predictors of mortality)	54 (17%)	OR = 2.72, *p* = 0.031	∗	∗	∗	∗
Almazeedi, S. et al.	July 2020	Kuwait	Retrospective cohort	1096	44 (4%)	OR = 10.09, *p* = 0.032	∗	OR = 5.86, *p* = 0.015	∗	∗
Ferrari, B.L. et al.	Jan 2021	Brazil	Longitudinal multicenter cohort	198	41 (21%)	OR = 3.4, *p* = 0.01	∗	∗	∗	∗
Wallis, T. et al.	Jan 2021	UK	Prospective single center cohort	101	35 (35%)	∗	∗	∗	∗	∗
*Preprints*										
Wang, A. et al.	June 2020	USA	Retrospective multicenter cohort	7,592	1,572 (20.7%)	aOR = 1.27, *p* = 0.041 (former); NS for current	∗	∗	∗	∗
Israel, A. et al.	June 2020	Israel	Case-control	24,906 (4,151 cases)	889 (21.4%) (cases)	NS	∗	∗	∗	NS
Khawaja, A. et al.	May 2020	UK	Prospective cohort	406,793 (605 with COVID-19)	331 (54%) (40k/10% current smokers in whole cohort)	∗	OR = 1.39, *p* < 0.001	∗	∗	∗
Romero, G.F. et al.	Nov 2020	USA	Retrospective cohort	577	268 (46.4%)	aOR = 1.99, *p* = 0.03 (former); NS for current	∗	NS	Former: 15.5% (*p* = 0.09), current: 19.8% (*p* = 0.022)	∗
Mendy, A. et al.	June 2020	USA	Retrospective multicenter cohort	689	170 (24.7%)	NS	aOR = 2.01, *p* < 0.001	aOR = 2.34, *p* = 0.01	∗	NS
Hasan, M. et al.	Jan 2021	Bangladesh	Prospective observational cohort	600	106 (17.6%)	∗	∗	∗	∗	∗

OR: odds ratio; aOR: adjusted odds ratio; HR: hazard ratio; RR: relative risk; NS: not significant; pkyr: pack years. ^∗^Not assessed.

## Appendix

### A. Search Terms

#### A.1. PubMed and LitCovid which Recognize Mesh Terms

(“COVID-19”[Mesh] OR “Coronavirus Infections”[Mesh] OR “SARS-CoV-2”[Mesh] OR “SARS-CoV-2” OR “SARS COV 2” OR “COVID-19” OR “COVID” OR “novel coronavirus”) AND (“Smoking Devices”[Mesh] OR “Smokers”[Mesh] OR “Tobacco Use”[Mesh] OR “Tobacco Products”[Mesh] OR “Tobacco Smoke Pollution”[Mesh] OR “Vaping”[Mesh] OR “Smoking” OR “tobacco” OR “vaping” OR “cigarette” OR “Juul” OR “smoke” OR “smoked” OR “vape” OR “tobacco use”) AND (“Mortality”[Mesh] OR “Hospitalization”[Mesh] OR “Outcomes” OR “Outcome” OR “mortality” OR “death” OR “hospitalization” OR “hospitalized” OR “morbidity” OR “complication” OR “complications”)

#### A.2. Scopus and Europe PMC (Limited to Preprints), which Do Not Recognize Mesh Terms

(“coronavirus infections” OR sars-cov-2 OR covid-19 OR covid OR “novel coronavirus”) AND (smoking OR tobacco OR vaping OR cigarette OR juul OR smoke OR smoked OR vape OR “tobacco use”) AND (mortality OR hospitalization OR outcomes OR outcome OR death OR hospitalized OR morbidity OR complication OR complications)
